# Ultrasound Characteristics of the Cavitary Corpus Luteum after Oestrus Synchronization in Heifers in Relation to the Results of Embryo Transfer

**DOI:** 10.3390/ani11061706

**Published:** 2021-06-07

**Authors:** Bartłomiej Maria Jaśkowski, Hartwig Bostedt, Marek Gehrke, Jędrzej Maria Jaśkowski

**Affiliations:** 1 Department of Reproduction and Clinic of Farm Animals, Faculty of Veterinary Medicine, Wroclaw University of Environmental and Life Sciences, 50-375 Wrocław, Poland; 2 Clinic for Obstetrics, Gynecology and Andrology of Large and Small Animals with Veterinary Ambulance, Justus-Liebig-University, 35392 Giessen, Germany; hartwig.bostedt@vetmed.uni-giessen.de; 3 Department of Clinical Sciences and Diagnostic, Institute of Veterinary Medicine, Faculty of Biological and Veterinary Sciences, Nicolaus Copernicus University, 87-100 Toruń, Poland; gehrke1@o2.pl (M.G.); jedrzej.jaskowski@gmail.com (J.M.J.)

**Keywords:** corpus luteum, cavitary corpus luteum, compact corpus luteum, ultrasound examination, heifers, embryo transfer

## Abstract

**Simple Summary:**

In cattle there are physiologically two morphological forms of the corpus luteum: compact and with cavity. During ultrasound examination a fluid filled cavity is clearly visible. Routine ultrasound examination is performed—as a part of preselection—in embryo-recipient heifers/cows. Some vets eliminate recipients with cavitary corpus luteum from embryo transfer, considering them to be less suitable for an embryo to develop. In our research, we showed that the level of progesterone in the blood of recipients on Embryo Transfer day was higher in the case of cavitary corpus luteum than in the case of compact ones. Similarly, the pregnancy rate of recipients with a cavitary corpus luteum was higher. The presence of cavity inside the corpus luteum should not be a reason for the elimination of recipients from embryo transfer. Moreover, cavitary corpus luteum may be a valuable indicator of the recipient’s reproductive potential.

**Abstract:**

The aim of the study was to conduct an ultrasound analysis of quantitative parameters of the corpus luteum (CL) in recipient heifers on days 6–8 after oestrus, and to compare reproduction potential of both types of CL in those females. Analyses were performed on 300 heifers, synchronized with two injections of cloprostenol. Clinical and ultrasound examinations of ovaries were performed and measurements of the CL were recorded. The blood samples were taken to determine progesterone level. Pregnancy examination was conducted after 6–8 weeks from the ET. Cavitary CL was found in 32.7% heifers In 48.0% of the cavitary CL, its luteal tissue area was reduced by 14.3% compared to the compact CL, while 16.3% of the CL had luteal tissue reduced by more than 33.8%. Progesterone level in blood serum was higher in heifers with the cavitary CL (*p* < 0.001). Pregnancy rate was higher for females with a cavitary CL (52%) than those with compact ones (33%, *p* < 0.05). The ultrasound assessment of luteal tissue should be included in the evaluation of the functional status of the CL in ET-recipient heifers. The cavitary CL presence may indicate a higher potential of the recipient in maintaining the pregnancy.

## 1. Introduction

The clinical examination of ovaries to confirm the presence of the corpus luteum (CL) is one of the component procedures in the selection of recipients on the day of embryo transfer (ET). Since the embryo is transferred into the ipsilateral horn of the uterus, several studies have shown that a CL of at least 17 mm in its diameter [1] guarantees a satisfactory conception rate and maintenance of pregnancy [1,2,3,4]. In turn, there is a tendency for lower pregnancy rates in heifers receiving fresh embryos if their progesterone (P4) level was below 1 ng/mL, and for previously frozen embryos when the P4 concentration was less than 3 ng/mL [5]. Ultrasonography is increasingly applied in veterinary gynecology [3,6,7,8,9,10]. It provides an accurate description of ovarian structures, including the objective determination of the position, number, dimensions, and structure of the CL [6]. Several parameters of ultrasound measurements, such as the diameter of the CL, the area, and the volume of luteal tissue, correlate with the blood P4 level [1,2,6,11,12,13]. Thus, the ultrasound characteristics of the CL detected on days 6–8 after oestrus may be of significant importance for the selection of recipients for ET.

The echostructure of the CL and its morphological form do not always correlate with the results of pregnancy rate and the level of blood P4 concentration achieved by the females with different types of the CL. As has been well known for many years now, in cattle the CL can occur in one of two morphological forms: compact or with cavity [14]. The latter is often larger in its diameter. Thus far, no influence has been attributed to the pregnancy rates of females with a cavitary CL. However, some veterinarians have doubts on the quality of the cavitary CL and disqualify such embryo recipients from the ET procedure [12]. The presence of the cavity inside the CL is not a sufficient basis for the elimination of the embryo recipient from the transfer.

The aim of the study was to conduct an ultrasound analysis of quantitative parameters of the CL in heifers on days 6-8 after the synchronized oestrus procedure and to compare reproduction potential of females with both types of the CL during the day of ET.

## 2. Material and Methods

### 2.1. Animals and Oestrus Synchronization

Analyses were conducted in the years 2015–2017 on a population of 300 heifers of Polish black–white Holstein–Frisian breed, at the age of 15–19 months, weight 380–460 kg, with at least two oestrus at the regular time (18–24 days), that were not inseminated before. Animals were kept in a free stall barn in groups of forty, maintained and observed by one or two qualified workers. Oestrus was synchronized at 14-day intervals administering an intramuscular injection of 0.5 µg cloprostenol (2.0 mL of Estrumate, Intervet, Schering-Plough Animal Health, Warsaw, Poland).

### 2.2. Clinical and Ultrasound Examinations

Clinical and transrectal ultrasound examinations of ovaries were conducted in all heifers on days 7 after oestrus, respectively, 29, 201, and 70 of them were exactly 6, 7, and 8 days after heat. Thus, only the animals which could be potential recipients in the Multiple Ovulation and Embryo Transfer (MOET) programme were taken into consideration. Examinations were performed using a portable ultrasonograph (iSkan Draminski, Olsztyn, Poland) equipped with a 7.5 MHz linear transducer. Ovarian luteal solid structures or luteal structures with the cavity ≤ 20 mm and the luteinized wall > 3 mm were defined as the CL. Corpora lutea visible on the monitor screen were determined as compact (without) or cavitary (with the cavity), and their longitudinal, transverse, and diagonal parameters were recorded (original measurement panel in the equipment of iSkan ultrasonograph, Draminski, Olsztyn, Poland).

### 2.3. Embryo Transfer Procedure

Animals immobilized in the standing stock were administered an intramuscular injection of 10 mg of xylazine hydrochloride (0.5 mL of Sedazin, Biowet Puławy, Puławy, Poland). Quality and development stages of embryos were assessed with the use of the scale developed by the International Association for Embryo Transfer [15]. Fresh embryos of minimum good quality at the morula or blastocyst stage were randomly transferred in the shortest possible time to recipients that underwent initial selection, regardless of the morphological form of the CL. Embryo transfer was performed using a Wörrlein gun (Goldenpick type, Minitüb GmbH, Tiefenbach, Germany) placed in a sanitary plastic casing (Minitüb GmbH, Tiefenbach, Germany). The embryo was deposited in the horn adjacent to the ovary with the previously diagnosed CL.

### 2.4. Corpora Lutea Measurements

Averaged longitudinal and transverse measurements were used to assess the mean diameter (Ø_CL_) and to calculate the cross-section area (A_CL_) of CL. Additionally, the diagonal measurement, according to formulae described by Miyazaki et al. [16], was used to calculate its volume automatically (V_CL_). When a cavity was observed, its diameter, area, and volume were calculated following the principles established for measurements of the compact CL. The basic analysis was conducted on one CL from each female. In the case of a larger number of luteal structures, the analysis was conducted on the largest CL.

### 2.5. Progesterone Analysis

Blood samples for P4 concentration analysis were taken from the coccygeal vein of 41 heifers: 25 with compact and 16 with the cavitary CL. After collection into 5 mL tubes, samples were transported to the laboratory in a chilled form, where they were centrifuged, and serum was secured in eppendorf tubes and frozen in −20 °C for further analysis. Serum P4 concentration analysis was performed by radioimmunoassay using P4 125 104 I RIA kit (catalog number IM1188, Immunotech, Prague, Czech Republic) according to the manufacturer’s instructions. All samples were tested twice. Measurable ranges were from 0.1 to 100 ng mL^−1^. The intra- and inter-test variation coefficient was 6.5% and 8.1%, respectively.

### 2.6. Pregnancy Confirmation

All females that received an embryo were examined for pregnancy 6–8 weeks after ET. The examination was conducted palpatively by an experienced veterinarian and confirmed by ultrasound examination (7.5 MHz linear transducer, iScan, Draminski, Olsztyn, Poland) The ultrasound visualization of the fetal heartbeat was considered the final confirmation. Pregnancy rate was calculated as the ratio of pregnant females to all embryo recipients.

### 2.7. Statistical Analysis

Results were statistically analyzed, applying the univariate analysis of variance and Tukey’s test in order to identify homogeneous groups. For P4 concentration analysis, after univariate analysis of variance, a Sidak multiple comparison test was used. The differences in pregnancy rate of heifers with one of two types of the CL were analyzed by Chi square Paerson test. All calculations were performed with the use of the STATISTICA 7.1, StatSoft^®^PL software package (Kraków, Poland).

## 3. Results

### 3.1. Morphometric Parameters of CLs

A single CL on one of the ovaries was predominant (99.7%), and only in one female were well-developed CLs (20 and 21 mm in diameter) detected on each ovary. Out of 300 heifers, 32.7% (*n* = 98) had a cavity in their luteal structure (Table 1). The average diameter, cross-section area, and volume of the cavitary CL were larger than those of compact ones (*p* < 0.001). Dimensions of the CL with a cavity on days 6, 7, and 8 after oestrus were bigger than those of the analogous compact CL, with significant differences recorded in relation to days 7 and 8 (Table 2). More significant dimensions of cavitary CLs were also confirmed when comparing them with compact CLs both on the same and the opposite ovary (Table 3). However, lateralization (right or left ovary) had no effect on the size of the compact CL or with the cavity.

On days 6, 7, and 8 after oestrus, mean values of all quantitative parameters seemed to increase irrespective of the morphological type of the CL, although they did not differ statistically (Table 4) (*p* > 0.05). The tendency of the cavity to grow was observed at slight changes in the luteal tissue amount (*p* > 0.05). Differences in the cavity size and the amount of luteal tissue in the CL on days 6, 7, or 8 after oestrus were high. In, as a rule, bigger cavitary CLs, the cavity dimensions, area, and volume of luteal tissue were slightly bigger (*p* > 0.05). The relationship between the size of the CL and its cavity is described by the regression equation and coefficients of correlation and determination: yØ_CL_ = 17.1674 + 0.5631 × Ø_C_, R = 0.606 and R^2^ = 0.368 (*p* < 0.001) (no data in tables). The mean size of the cavity (diameter) grew on consecutive days; however, the amount of luteal tissue (its area and volume) did not change or decreased (Table 5). The compact CL had a lower growth rate than the cavitary CL (Table 2). The mean daily increment in the compact CL diameter between days 6 and 8 after oestrus was 0.65 mm compared to 1.3 mm in the cavitary CL. The intensive growth of the latter was correlated with the dynamic growth of the cavity of 1.9 mm/day (Table 5).

**Figure 1:**
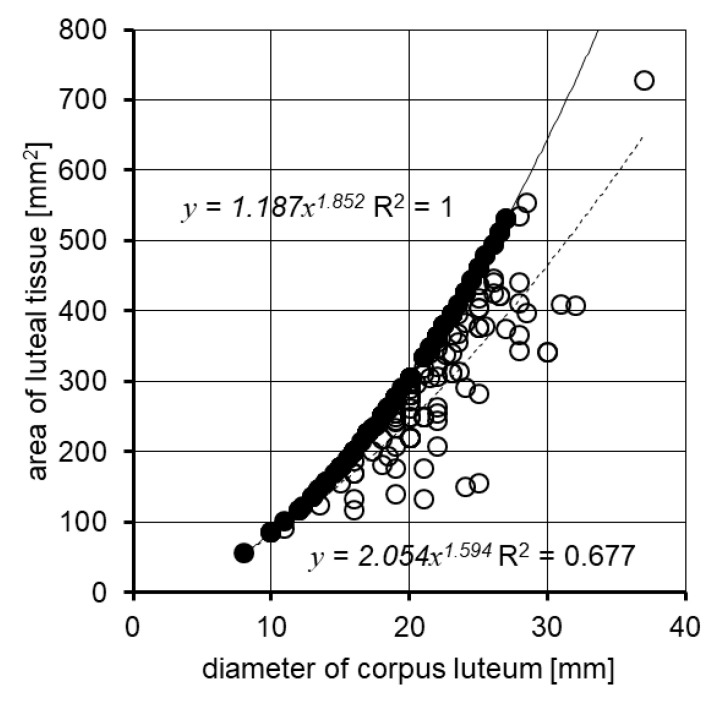
A comparison of area of luteal tissue in cavitary and compact CLs. ● area of luteal tissue in the compact CL; ○ area of luteal tissue in cavitary CLs. CL: corpus luteum.

**Figure 2:**
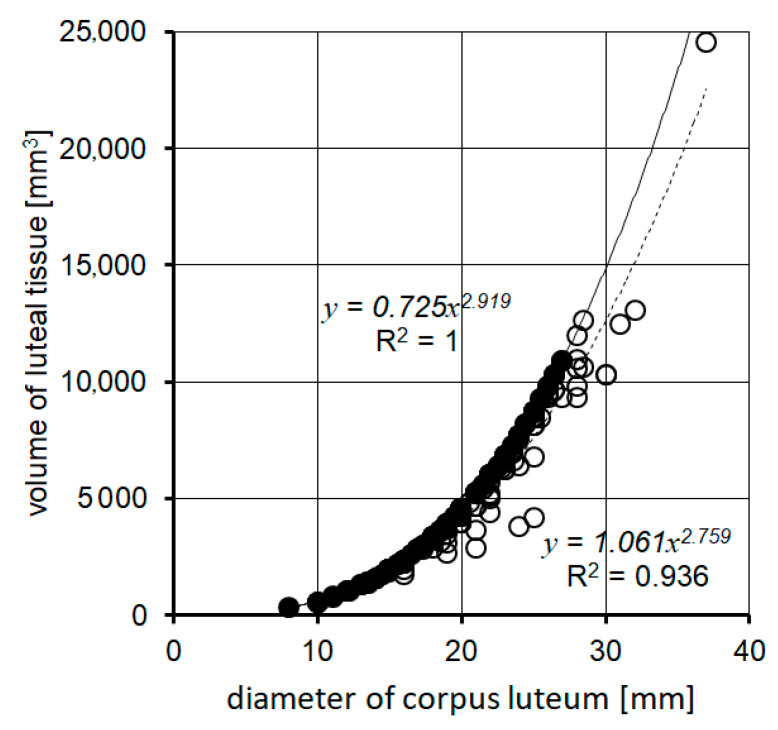
A comparison of volume of luteal tissue in CLs with and without a central cavity.● volume of luteal tissue in the compact CLs; ○ volume of luteal tissue in the cavitary CLs. CL: corpus luteum.

Figure 1 and Figure 2 present the luteal tissue area and volume variation in the CL with different morphological structures (compact and cavitary). They show that the amount of luteal tissue in the cavitary CL is smaller than its amount in the compact CL. Differences in the amount of luteal tissue in cavitary CLs of the same diameter are high. Correlations described are more visible when it comes to the luteal tissue surface area than its volume.

**Figure 3:**
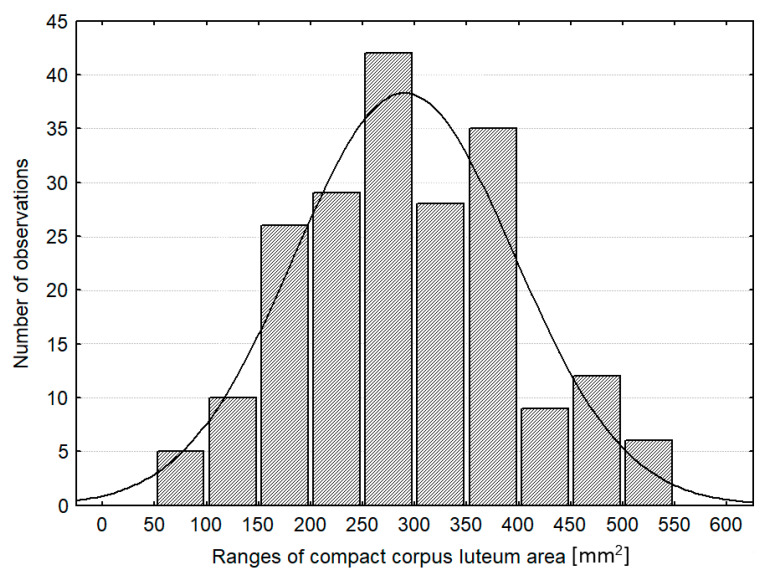
The number of observations in adopted size ranges of compact CLs; CL: corpus luteum.

**Figure 4:**
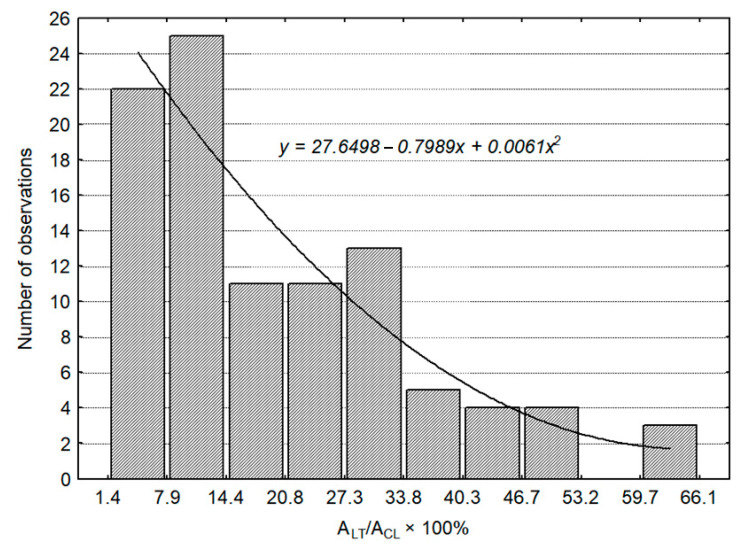
Variation in the range of luteal tissue losses in cavitary CL (in %) *n* = 98. A_LT_: area of luteal tissue; A_CL_: area of cavitary CL; CL: corpus luteum.

The recorded area of compact CL values had a normal distribution (Figure 3). Based on this, 6 (out of 12) the most numerous groups of similar diameters of CLs were established to fall within 14.3–23.8 mm. Corpora lutea with a size in this range were considered as “normal”. The corresponding ranges for area and volume of the CL were 163 mm^2^ and 1.659–7.588 mm^3^, respectively. They were calculated from regression equations, describing the dependence between the diameter, area, and volume of the CL (see: the footnote under Table 6). The percentage loss of luteal tissue resulting from the presence of the cavity was calculated for the cavitary CL. It was expressed by comparing the diameter, area, and volume of the cavity with corresponding values of the whole CL. The results are presented in Figure 4 and Table 7. In 48.0% of the cavitary CL, the luteal tissue area was reduced only by 14.3% in comparison to the area of luteal tissue in the same size of the compact CL, while 16.3% of the CL had luteal tissue reduced by more than 33.8%. Comparable boundary values of loss luteal tissue for the diameter, area, and volume of the cavity were calculated from regression equations given in footnotes under Table 7.

### 3.2. Serum Progesterone Concentration

**Figure 5:**
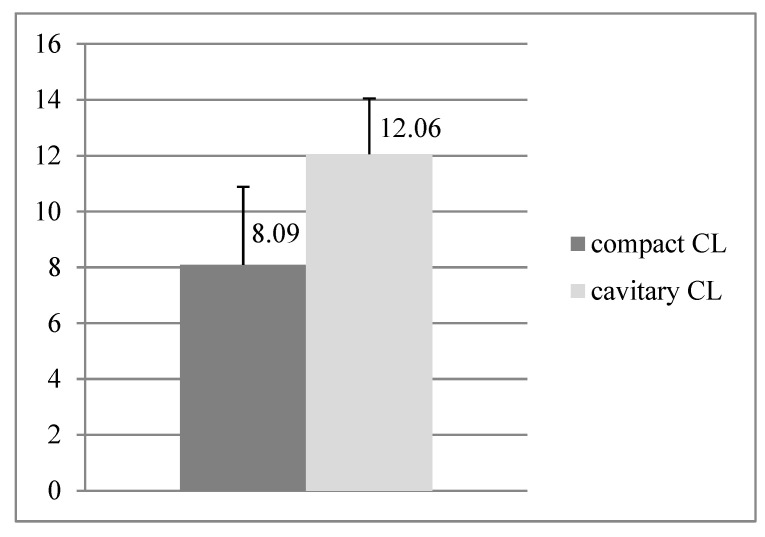
Comparison of P4 concentration in serum (ng/mL) of embryo recipients with the compact CL (*n* = 25) and cavitary CL (*n* = 16); CL: corpus luteum.

Serum P4 levels of heifers with the compact or cavitary CL on the ovary were significantly different (*p* < 0.001), while for the compact CL the mean amount of P4 was 8.09 ng mL^−1^, and for the cavitary CL the mean amount was 1.5 times higher—12.06 ng mL^−1^. Analysis of P4 concentration in the blood is presented in Figure 5.

### 3.3. Pregnancy Rate

The total pregnancy rate in embryo-recipient heifers was 39%, of which for females with the compact CL it was 33%, and for those with the cavitary CL it was 52% (*p* < 0.05). For the recipients animals on individual days of the cycle (6–8), significant differences between the CLs were not found on day 6: 45% compared to 44% for the compact and cavitary CL, respectively. On the remaining analyzed days, the recipients with the cavitary CL showed a significantly better pregnancy result: 50% vs. 34% on day 7, and 59% vs. 23% on day 8 (*p* < 0.05) compared to the compact CL. More detailed information can be found in Table 8.

## 4. Discussion

The mean size of the CL detected on days 6–8 after oestrus had a similar dimension irrespective of the number of days after oestrus (i.e., days 6 to 8) or their location (right/left ovary). The CL observed on day 6 should be slightly smaller than those observed on successive days. The probable cause for lack of differences (among two types of the CL) on day 6 could be due to the limited number of observations (29 vs. 201 and 70 for the other days). A bigger size of the CL on consecutive days may also suggest the necessity to determine individual criteria of their assessment on each of these days separately. Significant differences in quantitative parameters of the CL were determined first of all by the structure of their parenchyma. Cavitary CLs were larger than compact ones. Several authors indicated the presence of a small or large cavity in the ovarian parenchyma [2,6,17,18,19,20]. It has been also noted that the pregnancy probability was affected by the diameter of the CL, not by the P4 concentration [20]. Conversely, significant correlations between the size of the CL and P4 concentration were also noted before [21].

The frequency of the CL with the cavity incidence was 32.7% in our study, and it was comparable to that reported by others, e.g., 37.2% [19]. In relation to the size of the cavity, Kito et al. reported a frequency of 30.8% in the case of the CL with a cavity of minimum 7 mm in diameter and 24.4% with a cavity of minimum 10 mm. The echotexture of luteal tissue in the CL with and without the cavity was similar [19]. It was comparable to reports of other authors [22]. According to some studies, it undergoes evolution and involution proportional to development and regression of the CL [23]. In the cyclically growing, fully developed, and regressive CL, the cavity was observed in 42.1, 33.7, and 11.1% cases, respectively, while it was found in 5.1% of CLs graviditatis [14].

In this study, discrepancies were observed between dimensions of the CL and the size of their cavity. Relatively large cavitary CLs in different cavity sizes were found. However, as a rule, large cavities were observed in large CLs. Occasionally, such structures are considered to be ovarian cysts, and such potential ET-recipient heifers are unfitted. It can be seen from a study by Grygar et al. [24] that the cavitary CL may have a larger volume of luteal tissue and a higher secretion activity than the compact CL. In the same research, mean concentrations of P4 in peripheral blood in pregnant cows bearing a homogeneous CL or cavitary CL on day 9 were 3.15 and 4.12 ng mL^−1^, respectively. Concentrations of P4 were higher in pregnant in comparison with non pregnant females. Our results of P4 levels in heifers with a compact and cavitary CL present much higher amounts of P4 in cases with a cavity inside the CL (*p* < 0.001). It is in agreement with Grygar et al.’s [24] suggestions that cavity may have a positive effect on the function of the CL.

In our study, the cavity diameter was never greater than 2 cm, and luteal tissue width was >3 mm. In several cases in which cavitary CLs were found, in terms of their diameter, they had no equivalents in the compact CL, and their volume of luteal tissue considerably exceeded that recorded in the largest CL without the central cavity. However, the mean period for returning to oestrus and mean pregnancy rate noted by others [25] were not significantly different (*p* > 0.05) for cows that had the CL with or without the cavity.Moreover, cows with the cavitary CLs had a significantly higher albumin level, suggesting the metabolic effect on the formation of such a structure [25]. Furthermore, a tendency was observed for the incidence of the cavitary CL when the ovulatory follicle was larger (19 vs. 17 mm) [25].

Morphological and morphometric variability of the CL provided the basis for our proposal of clinically applicable selection criteria for ET recipients. As indicated by the observations in our study, the morphological type of the CL is determined (absence or presence of a cavity). In relation to the compact CL, diameter or its derivative, i.e., area or volume may be used. Depending on our criteria (see Table 3), they may be classified as small, normal, or large. Regarding the cavitary CL, their evaluation consists of the determination of the range for their diameter reduced by the cavity diameter or the area or volume of luteal tissue. The evaluation of the cavitary CL includes an additional parameter, i.e., the size of the cavity in relation to the total CL size expressed as a percentage. In this case, measurements of the diameter, area, or volume may be applied, qualifying them to a group with a minimal, average, or considerable loss of luteal tissue (criteria and denotations see Table 7). Clinical applicability for the described evaluation method of ET-recipient heifers and an indication of markedly disadvantageous variants require predictability of the model under field conditions, including the development of early pregnancy and the level of P4.

Among parameters used in the evaluation of the cavitary CL, we need to mention the duality of the evaluation of luteal tissue. Apart from the measurement of its amount based on the calculated area and volume, there is a ‘hypothetical’ potential for the qualitative evaluation by the comparison with the total CL size. At least three observations seem to indicate the accuracy of such inference.

Firstly, the growth rate of the cavitary CL is faster than that of the compact one. Secondly, in the cavitary CL of a comparable size, a considerable variation is observed in the amount of luteal tissue. Thirdly, the rate of the cavity growth on days 6, 7, and 8 exceeds that of the whole CL. Dynamic development of the CL may manifest primary disturbances in the development of the lutein cell layer (e.g., blood supply) or may be secondary in character (e.g., developmental disorders as a result of pressure caused by excessive accumulation of fluid). It is evident from studies that cavitary CLs are characterized by a larger content of type 1 lutein cells than compact CLs in cows with a fully developed cyclic compact CL [24,26,27,28]. However, as can be seen from the study by Perez-Marin [25], the fertility of cows with the CL containing the cavity did not differ from that of cows with the compact CL (42.9% vs. 57.1%, respectively). Moreover, the presence of the cavity in the CL after oestrus, i.e., when artificial insemination is performed, did not influence the expression of symptoms of estrogenization accompanying waves of follicle growth on the ovary, but it had a negative effect on conception rates [18].

The presence of the cavity and its size have been suggested not to influence the area of luteal tissue and P4 concentration [12,29,30]. In the case of bigger cavitary CLs, the area of luteal tissue was also bigger [31]. Moreover, in our study in exceptionally large cavitary CLs, the amount of luteal tissue was comparable with its amount observed in CLs of average size (e.g., >15 mm). We also found—which should be emphasized—the cavitary CL where the amount of luteal tissue was comparable with its amount in very small compact CLs (e.g., <15 mm). It was a slight percentage of CLs, hence lack of opinion on their influence on, e.g., early pregnancy development.

In our own study, we showed a relatively quick increase in the cavity and clearly slower growth of luteal tissue. Although we did not manage to confirm statistical differences among mean cavity sizes on consecutive investigation days (Table 4 and Table 5), their development was correlated with the development of the CL. The slower increase in the amount of luteal tissue may be the effect of dynamic development of the cavity and fluid fulfilling it, and the reason for the quantity of luteal tissue in the cavitary CL. They are differentiated by not only the size, the cavity size, and the amount of luteal tissue, but also the lack of luteal tissue resulting from the presence of the cavity. The last element of the evaluation of cavitary CLs has not been so far suggested as an additional criterion of morphological evaluation.

Pregnancy rate results after ET for females with the cavitary CL were significantly higher than in those with a compact CL. Until now, the prevailing view [3] was that in the case of ET, no differences were found in the effectiveness of both morphological types of the CL in recipients. Additionally, more recent studies of the cavitary CL [25] did not suggest a significant relationship between the morphological form of the CL and the pregnancy rate or indicated that the cavitary CL may have a negative impact on the pregnancy rate [32]. It should also be noted that the slightly lower overall pregnancy rate (39%) compared to that reported by other authors [3] could have been caused by a relatively late pregnancy examination. It is known that in cows there are cases of pregnancy loss, which in our studies, due to late pregnancy examination, were not reported.

## 5. Conclusions

In summary, the assessment of luteal tissue based on the morphometric ultrasound measurement should be included in the evaluation of the functional status of the CL in ET-recipient heifers. Our results show quite clearly that the cavity inside the CL is not the basis for disqualifying the recipient from ET. On the contrary, its presence may indicate a higher potential of the recipient to maintain the pregnancy. The higher concentration of P4 in the blood of recipients with a cavitary CL compared to heifers with the compact ones may also indicate a higher probability of pregnancy maintaining during the time of pregnancy recognition.

## Figures and Tables

**Table 1:** General characteristics of quantitative parameters of CLs on days 6–8 after oestrus.

Type of CL	n	Ø_CL_ Range (mm)	Ø_CL_ (mm)	A_CL_ (mm^2^)	V_CL_ (mm^3^)
Compact	202	8.0–27.0	19.1 ± 3.9 ^b^	288 ± 105 ^b^	4.424 ± 2.455 ^b^
Cavitary (cavity size)	98	11.0–37.0 (2.0–20.5)	22.1 ± 4.2 ^a^ (8.8 ± 4.5)	378 ± 135 ^a^ (103 ± 84)	6.757 ± 3.992 ^a^ (1.138 ± 1.264)
Total	300	8.0–37.0	20.1 ± 4.2	318 ± 123	5.186 ± 3.229

CL: corpus luteum; Ø_CL_: CL diameter; A_CL_: area of CL (with cavity); V_CL_: volume of CL (with cavity); ^ab^ *p* < 0.05.

**Table 2:** Comparison of CL parameters on days 6, 7, and 8 after oestrus.

Day after Oestrus	Type of CL	n	Ø_CL_ (mm)	A_CL_ (mm^2^)	V_CL_ (mm^3^)
6	compact	20	18.2 ± 3.5	263 ± 90	3.783 ± 1.829
	cavitary	9	20.7 ± 4.0	334 ± 124	5.545 ± 3.343
7	compact	139	19.1 ± 3.7 ^b^	287 ± 102 ^b^	4.379 ± 2.387 ^b^
	cavitary	62	21.8 ± 4.1 ^a^	369 ± 131 ^a^	6.474 ± 3.908 ^a^
8	compact	43	19.5 ± 4.5 ^b^	304 ± 121 ^b^	4.868 ± 2.836 ^b^
	cavitary	27	23.3 ± 4.4 ^a^	416 ± 145	7.810 ± 4.281 ^a^

CL: corpus luteum; Ø_CL_: CL diameter; A_CL_: area of CL; V_CL_: volume of CL; ^ab^ *p* < 0.001.

**Table 3:** Variability in parameters of CLs on the left and right ovaries on days 6–8 after oestrus.

Type of CL	n	Ø_CL_ (mm)	A_CL_ (mm^2^)	V_CL_ (mm^3^)
compact, right ovary	159	19.2 ± 3.9 ^b^	291 ± 104 ^b^	4.467 ± 2.416 ^b^
compact, left ovary	43	18.7 ± 4.1 ^b^	280 ± 111 ^b^	4.265 ± 2.618 ^b^
cavitary, right ovary (cavity size)	57	21.9 ± 4.2 ^a^ (9.1 ± 4.7)	371 ± 135 ^a^ (86 ± 81)	6.564 ± 3.846 ^a^ (845 ± 1.236)
cavitary, left ovary (cavity size)	41	22.5 ± 4.1^a^ (8.4 ± 4.2)	389 ± 136 ^a^ (73 ± 68)	7.025 ± 4.221 ^a^ (650 ± 955)

CL: corpus luteum; Ø_CL_: CL diameter; A_CL_: area of CL; V_CL_: volume of CL; ^ab^ *p* < 0.05.

**Table 4:** Comparison of cavity and luteal tissue parameters on days 6, 7, and 8 after oestrus.

Day after Oestrus	n	Ø_C_ (mm)	A_C_ (mm^2^)	V_C_ (mm^3^)	A_LT_ (mm^2^)	V_LT_ (mm^3^)
6	9	6.2 ± 1.9	37 ± 21	186 ± 177	297 ± 122	5.359 ± 3.334
7	62	8.7 ± 4.3	78 ± 71	708 ± 1.040	291 ± 102	5.766 ± 3.389
8	27	10.0 ± 5.3	102 ± 91	1.084 ± 1.397	313 ± 108	6.726 ± 3.391

Ø_C_: cavity diameter; A_C_: cavity area; V_C_: cavity volume; A_LT_: area of luteal tissue in ovary with cavity; V_LT_: volume of luteal tissue in ovary with cavity; *p* > 0.05.

**Table 5:** Comparison of cavity and luteal tissue with the total dimension of CLs on days 6, 7, and 8 after oestrus.

Day after Oestrus	n	Ø_C_ (% Ø_CL_)	A_LT_ (% A_CL_)	V_LT_ (% V_CL_)
6	9	30.5 ± 9.1	88.2 ± 7.0	96.1 ± 3.9
7	62	39.2 ± 15.6	80.2 ± 14.1	90.5 ± 10.6
8	27	42.1 ± 18.8	76.7 ± 16.9	87.6 ± 12.4
mean	98	39.2 ± 16.3	80.0 ± 14.6	90.3 ± 10.9

CL: corpus luteum; Ø_C_: cavity diameter; A_LT_: area of luteal tissue in cavitary CLs; V_LT_: volume of luteal tissue in cavitary CLs; Ø_CL_: CL diameter; A_CL_: area of CL together with a cavity; V_CL_: volume of CL together with a cavity; *p* > 0.05.

**Table 6:** Ranges of morphometric measurements of compact CLs determined based on the curve of normal distribution of their diameter (according to the curve from Figure 1, *n* = 202).

Type of Measurement:	Quality Classes of Corpora Lutea (Determined)		
	Small (L)	Normal (N)	Large (H)
Ø_CL_ (mm)	<14.3	14.3–23.8	>23.8
A_CL_ (mm^2^) ^1^	<163.9	163.9–420.4	>420.4
V_CL_ (mm^3^) ^2^	<1.659	1.659–7.588	>7.588
% observations in classes	9.4	77.7	23.8

CL: corpus luteum; Ø_CL_: CL diameter; A_CL_: area of CL; V_CL_: volume of CL; ^1, 2^ boundary values for A_CL_ and V_CL_ were calculated from regression equations: y_1_ = 0.611x^2^ + 3.721x − 14.29, R^2^ = 1 and y_2_ = 29.23x^2^ − 489.6x + 2683, R^2^ = 0.999, where y_1_: A_CL_, y_2_: V_CL_, x: Ø_CL_.

**Table 7:** Ranges of morphometric parameters of luteal tissue in the cavitary CL (according to the curve of distribution from Figure 4, *n* = 98).

Type of Measurement:	Categories of Luteal Tissue Losses in % (Determined)		
	Minimal (1)	Average (2)	Considerable (3)
^1^ Ø_c_/Ø_CL_ × 100%	<35.1	35.1–55.7	>55.7
A_c_/A_CL_ × 100%	<14.3	14.3–33.8	>33.8
^2^ V_c_/V_CL_ × 100%	<4.7	4.7–17.96	>18.0
% observations in categories:	48.0	35.7	16.3

CL: corpus luteum; Ø_C_: diameter of cavity; Ø_CL_: CL diameter together with a cavity; A_C_: area of cavity; A_CL_: area of CL together with a cavity; V_C_: volume of cavity; V_CL_: volume of CL together with a cavity; ^1, 2^ boundary values for ^1^ Ø_C_/Ø_CL_ × 100% and ^2^ V_C_/V_CL_ × 100% were calculated from regression equations: y_1_ = 0.070x^1.576^ and y_2_ = 8.319x^0.54^, where y_1_ is Ø_C_/Ø_CL_ × 100%, y_2_: V_C_/V_CL_ × 100%.

**Table 8:** Percentage of various morphological forms of the CL (6–8 days) after heat in pregnant and nonpregnant embryo-recipient heifers (*n* = 300; * *p* < 0.05).

CL Type	Morphological Diversity	Pregnant Heifers (%)	Nonpregnant Heifers (%)	*p*	Pregnancy Rate (%)
Compact CL	small	9.1	21.3	0.49	33
	normal	77.3	71.3	0.43	
	large	13.6	7.35	0.86	
Cavitary CL	minimal	49.0	53.2	0.77	52 *
	average	35.3	31.9	0.83	
	considerable	15.7	14.9	0.97	

CL: corpus luteum; *p*: (*p*-value) statistical differences between pregnant and non pregnant heifers.

## Data Availability

The datasets used during the study are available from the authors on reasonable request.

## Abbreviations

A_CL_	cross-section area of corpus luteum
CL	corpus luteum
ET	embryo transfer
Ø_CL_	diameter of corpus luteum
P4	Progesterone
V_CL_	volume of corpus luteum
